# The Czar’s Case

**Published:** 1855-07

**Authors:** 


*The Czar's Case.*—The great public, astonished to hear of the death of the Emperor of Russia, ere they knew of his illness, instantly concluded that an assassin's hand had given the peace to Europe which the arts of diplomatists and generals had failed to win. But the great public were altogether wrong, Europe is as far from peace as before, and the official bulletins and accounts received from various, and apparently unconnected sources, leave no doubt on the mind that the Czar died a natural death.

The following seems to be the most probable account of the course of his illness:

The Emperor had been for a considerable time in indifferent health, ready, if one may so say, to yield to any exciting cause of disease; mind and body had been over exerted. The powers of his nervous system had been stretched to the utmost. During the third week of February he began to suffer from influenza. He treated the ailment as of trifling import. On the 22d he was much worse; there was now a want of sleep and increased cough, with copious expectoration. In answer to the strongly-expressed opinion of Drs. Mandt and Karell that he would keep his room, Nicholas is reported to have replied, "You have done your duty, gentlemen, and I thank you, and now I wish to do mine," and to have at once entered his sledge and proceeded to inspect a body of guards. This, the 22d of February, was the last time he was seen in public, and he appeared, it is said, to all who saw him, to be evidently unwell; he coughed violently, and expectorated excessively. He observed to one of his attendants, as he left the exercising house—a place anything but warm—"I am in a perfect bath of perspiration." In the evening he complained of feeling cold, and kept his cloak on whilst in the Empress' room. He passed the 23d on the sofa, covered up with a cloak, and for the last time transacted some business. From the 24th to the 27th, official reports from the palace were, that "The Emperor does not leave his bed, as he is somewhat feverish; the cough is getting less and less hard."

On the night of the 28th February, the Emperor became rapidly worse. On the following evening, Drs. Mandt and Karell despaired of his recovery. During the night of the 1st, Dr. Mandt informed his patient of the serious nature of his indisposition. The Emperor answered very calmly, "And so you think that I am liable to a paralysis of the lungs?" to which Dr. Mandt answered, "Such a result is very possible." He enjoyed possession of intellect and speech up to the moment of his death, which took place without a struggle, in the presence of the whole family, at ten minutes past twelve o'clock, March 2d.

From this account it seems tolerably certain that the cause of the Emperor of Russia's death was capillary bronchitis, excited by exposure to cold while suffering from influenza. The depressing influence of the primary disease—influenza—on the powers of life is now universally admitted, and the frequency with which the disease is complicated by capillary bronchitis well known; the most common cause of death in influenza is, in fact, this secondary local affection.

Whether the disease would have proved fatal, had the Emperor been under the care of well informed practical physicians from the time that he kept his bed, is very doubtful. Dr. Mandt, in whose hands the Czar placed his life, is a homœopath, and judging from what we have seen and read of the treatment of disease by other homœopaths, his Majesty must have, from its outset, a very poor chance of surviving the secondary attack. Influenza occurring in a man so little advanced in life as was the late Emperor of Russia, would no doubt have terminated favorably had he taken ordinary care of himself, and that, whether he had swallowed Dr. Mandt's globules, or thrown them into the fire. But the case was very different when, through his own imprudence, severe capillary bronchitis had supervened; and then the most active medical treatment was necessary, and the highest skill and judgment required to direct it. To entrust the life of a man suffering from such diseases, to men ignorant enough to give to Europe the bulletins signed by Dr. Mandt, and to administer to him drugs in doses of a decillionth of a grain, was indeed to deprive him of all hope of recovery. The bulletins issued by Dr. Mandt and his colleagues, afford unmistakable evidence of the ignorance of their present state of pathological knowledge.—*Medical Times and Gazette.*

Did he die of inflammation and apoplexy of the lungs? If he did, we take upon ourselves to say that the course of the pneumonia was unusually rapid, and marked by circumstances inexplicable by the known laws of those affections. Dating the onset of the inflammation frum the 28th February, when pain in the right lung was observed, to the fatal conclusion on the morning of the 2d of March, it lasted but forty-eight hours at the utmost.

Or shall we surmise that the physicians had overlooked the earlier accession of one of the most severe of diseases? But, on the 1st of March, the second day of the supposed pneumonia, the fever had diminished and the expectoration was easier, and yet the pneumonia, if pneumonia there were, must have been progressing. These statements are utterly irreconcilable. With a pneumonia rapidly advancing to a fatal end, a pneumonia of only forty-eight hours' duration, the fever does not abate, and the expectoration does not become easier on the second day. In the early stages of inflammation of the lungs, the expectoration is always difficult and remarkably viscid. To reason medically, concerning a disease which has a remarkably definite course, it is then probable that on the second day of this rapid and suspicious illness there was no pneumonia at all. But then it is said that, at a period which we must presume to have been about twelve hours before death, "atrophy of the lungs was feared;" and some hours later, Dr. Mandt communicated to the Emperor "that atrophy was possible." Now what particular idea the public may entertain of atrophy of the lungs, we know not; but every medical tyro knows that atrophy is a chronic change of almost imperceptible advances, and that it forms no part of an acute pneumonia. If we are not at liberty to accuse the physicians of the Emperor of the grossest ignorance, we must conclude that the word "atrophy" is altogether a mistake, taken up by unprofessional persons. Was it apoplexy then, that destroyed the Czar? Apoplexy and inflammation of the lungs are not absolutely incompatible. But there is something reported which is absolutely incompatible with either or both of these conditions as related in this incomprehensible history. Apoplexy and inflammation of the lungs preclude the idea of the Emperor, in his last moments, blessing his wife, children, and grandchildren, separately, with a firm voice. We are then reduced to this alternative: either the symptoms reported are fabulous or imperfectly related, or pneumonia and apoplexy of the lungs did not cause death.—*Lancet.*

Imperial pulmonary apoplexy has during the last fortnight been the text of many discussions in professional and non-professional circles. Is the "imperial" a distinct species of pulmonary apoplexy hitherto unknown to pathologists, and as yet undescribed by any author? The signs of pulmonary apoplexy from mitral disease had never been shown, or at least had never been spoken of, in reference to the Czar; yet, while his body was yet warm, Europe was informed that the troubler of nations had suddenly died of this slow malady. A few days before his death, Nicholas was described as in health, portentously energetic, and unwearied in the pursuit of his ambitious schemes, presenting the image of some infernal king of men turning at bay against the world. Suddenly we were informed that he had died of a disease which, in order to be rapid at its close, must have been long known to his physicians, and which must have disqualified him for the great physical efforts he had been making during the preceding weeks. Of the various forms of pulmonary apoplexy, would any explain this sudden termination except that arising from encephaloid cancer, when the pleura is torn, and great hæmorrhage takes place, causing sudden death? This variety, says Dr. Walshe, in his "Diseases of the Lungs and Heart," is "decidedly rare." Its existence is also incompatible with the previous good health and energy of the Czar. If from mitral disease also, the symptoms would have been long manifest. Nor, so far as we know, is it impossible to pronounce that death has taken place from pulmonary apoplexy, resulting from regurgitation at the mitral valve, without a post mortem inspection of the body. This, when England first learnt the news, could not have taken place; nay, every capital in Europe was convulsed by the shock of the Czar's doom ere his corpse had become rigid in death.—*Association Medical Journal*, from *Charleston Medical Journal.*

